# Variability in Open-Field Locomotor Scoring Following Force-Defined Spinal Cord Injury in Rats: Quantification and Implications

**DOI:** 10.3389/fneur.2020.00650

**Published:** 2020-07-09

**Authors:** Nick D. Jeffery, Kiralyn Brakel, Miriam Aceves, Michelle A. Hook, Unity B. Jeffery

**Affiliations:** ^1^Department of Small Animal Clinical Sciences, Texas A&M University, College Station, TX, United States; ^2^Department of Neuroscience and Experimental Therapeutics, School of Medicine, Texas A&M University, Bryan, TX, United States; ^3^Department of Biology, Texas A&M University, College Station, TX, United States; ^4^Department of Veterinary Pathobiology, Texas A&M University, College Station, TX, United States

**Keywords:** reproducibility, BBB scale, behavioral, reference interval, function

## Abstract

Spinal cord injury research in experimental animals aims to define mechanisms of tissue damage and identify interventions that can be translated into effective clinical therapies. Highly reliable models of injury and outcome measurement are essential to achieve these aims and avoid problems with reproducibility. Functional scoring is a critical component of outcome assessment and is currently commonly focused on open field locomotion (the “BBB score”). Here we analyze variability of observed locomotor outcome after a highly regulated spinal cord contusion in a large group of rats that had not received any therapeutic intervention. Our data indicate that, despite tight regulation of the injury severity, there is considerable variability in open-field score of individual rats at 21 days after injury, when the group as a whole reaches a functional plateau. The bootstrapped reference interval (that defines boundaries that contain 95% scores in the population without regard for data distributional character) for the score at 21 days was calculated to range from 2.3 to 15.9 on the 22-point scale. Further analysis indicated that the mean day 21 score of random groups of 10 individuals drawn by bootstrap sampling from the whole study population varies between 9.5 and 13.5. Wide variability between individuals implies that detection of small magnitude group-level treatment effects will likely be unreliable, especially if using small experimental group sizes. To minimize this problem in intervention studies, consideration should be given to assessing treatment effects by comparing proportions of animals in comparator groups that attain pre-specified criterion scores.

## Introduction

Spinal cord injury research aims to understand the mechanisms of tissue destruction following trauma and to identify interventions that can be translated into effective therapies in the clinic. Essential prerequisites include reliable models of spinal cord injury and outcome analyses that allow highly reproducible discrimination of intervention effects. During the past two decades, introduction of computerized monitoring of actual applied force and its duration have refined spinal cord injury models (1–3), thereby minimizing variability in cord damage and providing groups of animals in which the signal of an effective intervention can be more easily discerned against the inherent noise of the biological system. There are many aspects to assessment of an intervention effect, including histologic and electrophysiological analysis, but behavioral function, such as voluntary limb movement and coordination, is a critical component outcome because it can provide persuasive evidence in favor of translatable effectiveness.

Currently, most spinal cord injury research focuses on rodent models, and a multitude of methods has been developed for functional outcome assessment following spinal cord injury (4). Since its introduction by Basso et al. (5), the open-field locomotor scoring system commonly referred to as the “BBB scale” has become widespread as a key method to assess hind limb motor function and coordination between limb girdles after thoracic spinal cord injury. This method has the advantage of requiring no specialized equipment and being simple to apply, allowing large numbers of animals to be rapidly evaluated. The correlation between the open-field score and severity of histologic injury has been established (5, 6) and the scale also demonstrates high inter-rater repeatability (5, 7). The BBB scale is ordinal; locomotor function is categorized into ordered groups and so is not truly numerical (i.e., the difference in function between scores 1 and 3 cannot be considered equal to that between 13 and 15 for instance). This has led some authors (8) to question whether it is appropriate to analyze the resulting data using parametric statistics (because these methods assume that the data are numeric and normally distributed). On the other hand, others have argued that, because of the numerous categories, the error made in assuming a normal distribution makes little practical difference (9) or can be largely circumvented by modifications of the scale (10).

Although spinal cord injury models have become much more sophisticated recently and produce highly reproducible lesions, the variability in outcome between individuals, whether with or without experimental intervention, has received relatively little attention. Although examination of group-level difference clearly has precedence when analyzing the effects of an intervention applied to one of two groups of spinal cord-injured animals, there are also many benefits to examining individual-level effects. First, variability within and between individuals provides a background upon which to understand the magnitude of an intervention effect. If there is considerable spontaneous variability between animals then it can lead to both type I and type II erroneous conclusions regarding efficacy. Quantification of variability also allows its identification as a possible cause for lack of reproducibility, which has been highlighted as a problem in neuroscience generally, including spinal cord injury research (11). Second, patients living with spinal cord injury need to know how much benefit they might attain from an intervention and how likely they are to achieve such benefit. These are not questions that can readily be answered from group-level data.

Many measurements made in neuroscience, including many of those used for quantifying outcome in spinal cord injury experiments (12), create datasets in which sources of variability can be partitioned to provide outcomes that can be interpreted at both group- and individual- level (13). Unfortunately, the BBB scale (5) presents two major obstacles to analysis by this method. First, there is a “ceiling effect” in that normal animals show little variability on this scale and will almost invariably score at the top boundary of the range [i.e., (14)]; and second, the scale is ordinal despite being presented as numbers. The ceiling effect prohibits analysis of variability in normal animals (which are used to derive “reference change values”) and the ordinal nature of the scale implies that derived standard deviations, which are essential for partitioning analysis, do not have the same meaning at all score values.

In this report we describe an alternative approach to quantify variability in open-field locomotor scoring following a standardized spinal cord contusion and show how this information can be used to aid interpretation of outcomes after experimental spinal cord injury and improve experimental design. We reasoned that by analyzing BBB scores of a large number of rats after a defined severity of spinal cord injury we could construct a reference interval of the values that can be expected at specific time points. In clinical laboratory medicine, reference intervals define the boundaries within which 95% of the measured values of a specific analyte, for example a blood component, will fall, meaning that values outside these limits can be flagged as unusual. There are well-established rules for establishing these boundary limits and the most effective and representative are those derived by non-parametric or bootstrapped methods, because they do not depend upon assumptions that may be unrealistic or inaccurate regarding data distribution (https://clsi.org/media/2458/ep28a3ce_sample.pdf).

## Methods

In this study we aimed to quantify the variability associated with a standardized spinal cord injury and BBB scoring applied over a 21-day follow-up period. The subjects for analysis were rats that were controls in previously published experiments (15–17), or will be included in future publications, and had undergone a moderate T12 spinal cord contusion but had not received systemic or intraspinal medications, nor spinal implants. Open-field scoring was carried out by investigators trained and experienced in the technique and blinded to treatment allocation. All experiments were conducted according to national guidelines and Institutional Animal Care and Use Committee approval under a specific Animal Use Protocol.

Subjects were ~3-month old male Sprague Dawley rats (300–350 g) at the time of spinal cord injury, which was induced using standard techniques (15–17). Briefly, each rat was anesthetized using 5% isoflurane in oxygen and maintained at a concentration of 2–3% during surgery. The spinal cord at T12 was exposed via laminectomy, leaving the dura mater intact. The Infinite Horizons Impactor (Precision Systems Instrumentation) was fixed to the vertebral column and a moderate injury of 150 kdyne with a 1-s dwell-time was applied. The wound was closed with Michel clips and each rat received subcutaneous saline and antibiotic by injection. Michel clips were removed 14 days after surgery.

Before surgery, each rat was acclimated to the open-field scoring area—an open enclosure of 99 cm diameter and 23 cm deep—for 5 min per day for 3 days. After surgery, locomotor function was assessed for 21 days using the BBB scale; scores were recorded once daily by a blinded investigator on days 1, 2, 3, 4, 5, 6, 7, 9, 11, 13, 15, 18, and 21. If the scores for the right and left hind limbs differed the mean value was recorded.

Several analyses were applied. First, the raw data was plotted to summarize changes in locomotor function in the group as a whole. To investigate the variability in recovery between rats we then plotted the BBB score of each individual at day 1 and day 21. As a measure of variability we then calculated the bootstrapped reference interval (the range of scores within which 95% of the values fall) for BBB score at day 21, including the 90% confidence intervals for those estimates. This method of reference interval does not depend upon assumptions about data distribution. The same reference interval analysis was then repeated after the data had been transformed according to the conversion method described by Ferguson et al. (10) and, also, for a modified subset of our data from which animals with extreme BBB scores recorded at day 1 (i.e., those scoring <1 or more than 10; or, alternatively, those scoring more than 8) had been removed [because this is sometimes used with the aim of limiting variability, see (18)]. Lastly, 100 virtual “groups” of 10 rats were created by bootstrap sampling of the entire population and the mean day 21 score of each virtual group was calculated so as to generate a range of mean group values that could be expected to arise through chance alone.

Data analysis was carried out using Excel, GraphPad Prism and Stata 14 (Stata for Windows, StataCorp, College Station, TX).

## Results

**Figure 1:**
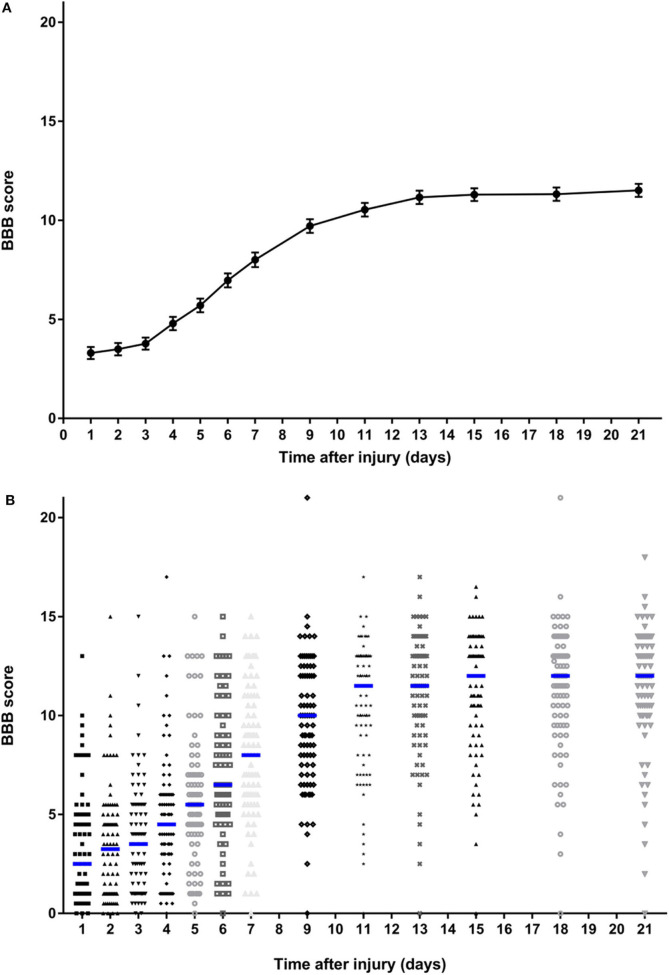
Summary data on BBB scores of all rats over the 21-day follow-up period following standardized 150 kdyne contusion injury at T12. **(A)** Points represent means and bars indicate standard error of the mean (s.e.m.) at each time point. **(B)** Dotplot showing score for rat at each time point; median at each time point is indicated by a blue bar.

A total of 86 rats were included in this study. As expected, the group-level summary BBB data produced a sigmoidal curve (Figure 1A), with a short lag phase between 1 and 4 days, a more rapid phase of recovery between 4 and 13 days, followed by a functional plateau between 15 and 21 days. The entire plot and the mean value achieved at day 21 are very similar to those previously reported following the same severity of injury (3, 19, 20) applied at nearby spinal cord segments. The error bars (s.e.m.) are small (because of the large population) and this graph does not fully illustrate the variability that exists within these data, whereas the dotplot shown in Figure 1B shows this in more detail. While there is a clear trend to recovery of function within the group as a whole, there is considerable variation in BBB score between animals at each time point and, importantly, a clearly left-skewed distribution. Although these data were derived from control animals from a series of experiments, the surgery was carried out by the same investigators who had trained in the same laboratory and the experiments followed sequentially during a period from 2017 to 2019. Kruskal-Wallis testing did not detect difference in day 21 scores (*H* = 3.39; *P* = 0.50) between the animal groups created for the original experiments [including data published in (15–17)].

**Figure 2:**
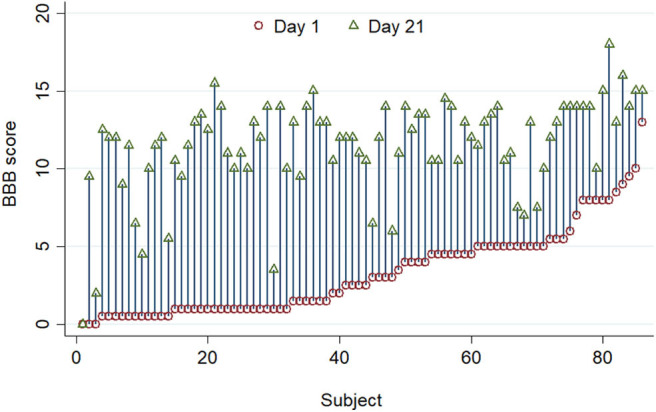
A standardized 150 kdyne T12 contusion injury was created in 86 rats, and this graph summarizes BBB scoring performed on day 1 (red circle) and day 21 (green triangle) after injury. Individuals are ordered by their day 1 scores. There is considerable inter-individual variation in the improvement in BBB score after injury.

There was also considerable variability in recovery between individuals (Figure 2). There is a clear visual trend that animals with more severe initial functional loss tend to have a worse final score at day 21, which is confirmed by analysis of regression of day 21 scores on day 1 scores (β = 0.462, 95% CI: 0.244–0.679; *P* < 0.001) and has been previously reported (21). From these data the calculated reference interval for day 21 scores is shown in Table 1, which, in this large study population that has a clearly left-skewed distribution (Figure 1B) was derived by bootstrapping (thus avoiding the problems associated with deriving a reference interval from standard deviation which inevitably assumes a normal distribution). This analysis implies that, following a 150 kdyne injury at T12 as applied here, it is to be expected that 95% of rats will score between 2.3 and 15.9 at day 21; in practical terms, 2.5% of rats will score 2 or less, and 2.5% will score 16 or more, at day 21.

**Table 1:** Summary of bootstrapped reference intervals for day 21 BBB scores derived from the entire cohort, or selected sub-groups following 150 kdyne injury at T12.

**Analyzed population**	**Lower bound (90%CI)**	**Upper bound (90%CI)**	**Comments**
All rats (*n* = 86)	2.3 (0–5.1)	15.9 (14.2–17.6)	Only scores of 2 and less or 16 and more can be considered an “unusual” recovery.
All rats, except those scoring <1 or >10 on day 1 (*n* = 70)	5.4 (3.3–7.6)	16.5 (14.7–18.2)	Expands “space” at lower end of the scale, so more readily detects animals with poor function.
All rats, except those scoring >8 at day 1 (*n* = 81)	2.1 (2.5–8.5)	15.5 (13.4–17.6)	Makes no practical difference compared with inclusion of all animals.
All rats—Ferguson et al. (10) score conversion (*n* = 86)	2 (0.4–3.6)	12 (12)	Upper bound corresponds to “ceiling” score, implying poor sensitivity for interventions that improve outcome after a lesion of this severity.

CI, confidence interval. Confidence intervals are much wider for the lower bound than the upper bound, which is a consequence of the left-skewed distribution. This result implies less confidence in the certainty with which values can be designated “unusually low” compared to “unusually high.”

Exclusion of specific individuals with extreme scores soon after injury has been suggested as a means to reduce variability in outcome at later time points (18) and we have calculated modified reference intervals to examine the effect of such exclusions. In our dataset, exclusion of rats that score either <1 or 10 or more at day 1 generates a modified reference interval for day 21 scores of 5.4–16.5. In practice, this implies that a rat would have to score 5 or less or 17 or more to fall outside these modified reference interval boundaries. Alternatively, exclusion of rats scoring more than 8 on day 1 results in a reference interval for score at day 21 that differs little from that calculated when all animals are included (boundaries are 2.1 and 15.5) (Table 1). Finally, transformation of raw data by the method described by Ferguson et al. (10), produces a corresponding bootstrapped reference interval from 2 to 12; 12 is the maximum score on this scale.

Finally, we determined the range of day 21 mean BBB scores that may arise through chance alone for groups of 10 untreated rats following this severity of injury in this laboratory. A group size of 10 was selected to approximate the sample size typically used in spinal cord injury research (14) and we generated 100 of these “virtual” groups through bootstrap sampling of the entire dataset. The lowest and highest mean scores amongst these 100 groups of 10 rats were 9.5 and 13.5, respectively.

## Discussion

BBB open field scoring is a key component of experimental spinal cord injury models and understanding variability in this outcome is critical for interpretation of intervention effects. Here we provide two analyses demonstrating that BBB scores at day 21, a point at which group scores reach a plateau, vary considerably even amongst rats that sustained a standardized injury of specific severity and received no intervention. We show that there is a wide reference interval, implying a wide range of potential scores and, in support, bootstrapped re-sampling of our dataset implies that a 4-point difference in mean values between experimental cohorts of 10 rats can arise purely through chance. It is important to stress that, although our calculated reference intervals may appear wider than expected, routine analysis of our dataset reveals a calculated standard deviation of ~3 at day 21, which is similar to that reported elsewhere for similar lesion models (3, 19, 20). In those experiments the reported standard deviation of ~3 implies a 95% reference interval of ~12 points on the BBB scale (i.e., an interval of 1.96 standard deviations above and below the mean value), which differs little from our calculated bootstrapped reference interval of 13.6 points. The two methods of calculating the reference interval differ because of the assumption of a normal distribution implicit in the calculation of standard deviation and the lack of this assumption in our bootstrapped calculation of the reference interval. As is shown in Figure 1B, the data are clearly not normally distributed but have a left-sided skew.

Whilst group-level analysis of intervention effects must always predominate, recognition of individual variability may have important implications for design and interpretation of functional outcome data, especially when considering how to translate experimental interventions from laboratory to clinic. First, as in any experiment, excessive outcome variability will blunt the ability to detect differences between groups because it will impair discrimination of an intervention effect—the signal of which will inevitably be less easily discerned amongst the noise of variability. Second, the magnitude of an intervention effect at group level can be put into context by comparing it with the width of the reference interval as a whole. A mean difference between groups that is much smaller than the width of the reference interval might be biologically interesting but may be of questionable translational importance [this distinction is similar to those made between explanatory and pragmatic clinical trials (22, 23)]. Similar conclusions might also apply when comparing an intervention effect with the 4-point difference that can arise spontaneously between randomly sampled groups of 10 injured but untreated rats.

Although BBB scale results are routinely reported as a group mean value (with standard deviations) the reason for differences between groups is in reality due to differences in the proportion of rats that fall into the specific ordinal categories. As previously highlighted (24, 25), a difference in mean value between intervention and control groups may result from a small response in many individuals or, alternatively, from lack of response in most individuals combined with a large response in a small proportion. In medicine, changes in analyte values that lie wholly within reference interval are usually ignored because of their lack of clinical importance. Therefore, in laboratory studies that aim to have translational impact it is highly advantageous to identify specific individuals that show exceptional outcome. If such individuals constitute a large proportion of an intervention group it provides strong evidence of translatable effect. Furthermore, investigation of reasons for that exceptional response can be helpful to direct further research. In this study, by determining the reference interval for outcome at day 21 we place boundaries on what might be considered “normal recovery” after the lesion at this site and of this severity. The finding that the day 21 reference interval, consisting of 95% of BBB scale scores, lies between 2.3 and 15.9, implies that only quite extreme BBB scores (of 16 or more, or 2 or less) could be regarded as unequivocal evidence of an exceptional intervention-driven effect in a single individual.

Our findings also have implications for experimental design. The wide reference interval for scores at day 21 following this specific injury suggests that it might be difficult to detect a detrimental effect of an intervention: because the lower reference interval boundary is 2.3 there is not much “space” for animals to exhibit unusually poor scores. This might be solvable by simply extending the experiment for longer to allow for further recovery, although the group-level data (Figure 1A) suggest a plateau is reached by day 21. Alternatively, if a detrimental effect were sought, a less severe injury might be induced, or the analyzed population might be altered by removing animals with more extreme values at day 1, as shown in Table 1. Similarly, although previously-recommended data transformation might improve the nature of the data in terms of statistical analysis (10), the reference interval for day 21 scores on this modified scale implies that a ceiling is reached in our data, suggesting that using this severity of injury and this scoring scheme might make it difficult to detect a beneficial intervention effect.

Our analysis also suggests an alternative approach to using the BBB scale for examining group effects of interventions. Rather than comparing groups by using mean and standard deviations, investigating intervention effects by comparing the proportions of animals reaching a specific criterion on the BBB scale may be advantageous, especially when considering translation potential. Spinal cord injury patients wish to know how much they are likely to benefit from an intervention and how likely that is to happen, both of which cannot be answered by comparing group means. In contrast, if a reference interval for outcome at a specific time after injury is known and an intervention is shown to improve the function of a large proportion of rats beyond that reference interval, then both those questions are addressed. Although it might not be the most efficient first step in pre-clinical investigation of intervention effectiveness, attainment of defined criteria might be a highly effective screening process to identify interventions to be taken forward for clinical translation.

Using a proportion of subjects that reach a (pre-defined) criterion is straightforward for sample size calculation, but might lead to an increase in the number of rats required for this type of pre-clinical testing. For instance, in the data that we present here, 5% of rats achieve a score of 15 or more at day 21 (Figure 1B). If we were to state that we wished to determine whether an intervention might increase that incidence to 30% we would require a sample size of about 35 rats per group [see MedCalc.net or (26)]. Such a change in outcome would be strongly suggestive of translational potential since it would represent a major change in function for a large proportion of affected rats.

### Limitations

Finally, we stress that we are presenting these data to illustrate a principle in data analysis, rather than to imply that our results can be directly transferred to other laboratories for outcome inference. Our analysis does not elucidate the origin of the variability, which may arise from variation in severity of injury, in each animal's expression of disability, or in investigator scoring of disability. For instance, although lesions produced by the Infinite Horizons device are reproducible (3) there might be subtle differences, perhaps associated with minute differences in impact position, in the severity of injury induced by investigators in other laboratories even when using the same impactor device and impaction variables. Furthermore, uncontrollable variables, such as the phase of the heart beat (i.e., systolic vs. diastolic pressure) at the instant of impact, imply some inevitable variability in tissue injury.

All these factors contribute to limitations in generalizing our results to apply to data from other laboratories. While there is evidence of transferability of BBB scoring between laboratories (7), it is important to note that the preparation and handling of the animals before and during testing may also have effects on the ascribed scores (27). As in hospital laboratories, it is imperative to reduce this “pre-analytical variation” as far as possible (28). Nevertheless, the magnitude of variability may differ in the hands of other researchers, with lesions of different severity or if outcome is assessed at later follow-up. Generally, for instance, function after spinal cord injury gradually improves and so its variability in a group of rats sustaining the injury used in this study might be much less at (say) 6 weeks than at day 21. This would be a very worthwhile subject for further study because, if such reduction in variability were to be defined, it would aid design of more efficient experiments to detect intervention effects: reduced variability enhances recognition of intervention signal vs. noise.

This brief report focuses on describing a bootstrap method to measure variability in outcome rather than discussing inter-group comparisons. The formal quantification of a reference interval in control animals is the first stage in developing the complementary individual-level analysis that we are proposing, because it identifies useful threshold criteria to indicate “exceptional” outcomes in untreated animals. The next stage would be to run routine experiments in which outcomes in intervention and control groups are compared. Group means would be compared using standard methods and then this would be augmented by complementary analysis of the proportions of individuals that reach the pre-defined outcome criterion identified from the reference interval. For instance, using this lesion severity with outcome analysis at day 21, comparison (by χ^2^ or Fisher's exact test) of proportions of rats in each group attaining BBB score of 15 or more would be a useful complementary analysis. The bootstrap methods used in this report would not be required for such comparison studies. The variability of outcome in animals that receive an intervention may differ from that of controls, and while this is an interesting field for future investigation, it will not hinder comparison of proportions of control and intervention groups that attain specific outcome criteria.

Potential differences in both injury and scoring factors between laboratories imply that, similar to quality control procedures in hospital laboratories, each spinal cord injury laboratory would need to derive their own reference intervals for each level of injury severity. Although at first sight this might appear to present a daunting impediment to applying these methods, in practice it may not be too problematical because, as we show here, appropriate data from large numbers of control animals are often already available from previous experiments.

## Data Availability Statement

All datasets generated for this study are included in the article/Supplementary Material.

## Ethics Statement

This animal study was reviewed and approved by Institutional Animal Care Committee at Texas A&M University.

## Author Contributions

NJ, UJ, and MH contributed conception and design of the study. MA and KB carried out the experimental work and organized the database. NJ and UJ performed the statistical analysis. NJ, UJ, and MA wrote sections of the manuscript. All authors read and approved the submitted version.

## Conflict of Interest

The authors declare that the research was conducted in the absence of any commercial or financial relationships that could be construed as a potential conflict of interest.
